# Relationship between paravertebral muscle function, pelvic incidence, and health-related quality of life in patients with degenerative spinal deformity

**DOI:** 10.1186/s13018-024-04593-3

**Published:** 2024-01-31

**Authors:** Can Chen, Yong Tang, Sen Yang, Wei Dai, Jiulin Tan, Xueke Yu, Chengmin Zhang, Fei Luo

**Affiliations:** 1 Department of Orthopaedics, Southwest Hospital, Army Medical University (Third Military Medical University), 30 Gaotanyan Street, Shapingba, Chongqing, 400038 People’s Republic of China; 2 Department for Combat Casualty Care Training, Training Base for Army Health Care, Army Medical University (Third Military Medical University), Chongqing, 400038 People’s Republic of China; 3 Department of Orthopaedics, The 72nd Group Army Hospital, Huzhou University, Huzhou, 313000 Zhejiang People’s Republic of China

**Keywords:** Paravertebral muscle, Pelvic incidence, Health-related quality of life

## Abstract

**Background:**

Patients with degenerative spinal deformity often experience symptoms that seriously affect their quality of life, such as low back pain and dysfunction. This study aimed to investigate the relationship between paravertebral muscle function and pelvic incidence (PI) and their effect on health-related quality of life (HRQL) in patients with degenerative spinal deformity.

**Methods:**

A total of 112 patients with degenerative spinal deformity in Southwest Hospital (Chongqing, China) were enrolled. They were divided into groups according to PI angle: high (PI > 60°, *n* = 37), normal (PI 50°–60°, *n* = 31), and low (PI < 50°, *n* = 44). Paravertebral muscle strength and endurance were assessed using the prone external fixation test frame. The sagittal vertical axis (SVA) was measured on X-rays of the spine in an anterolateral position, and all subjects were assessed with the Oswestry Disability Index (ODI), Roland–Morris questionnaire (RMQ), and 36-Item Short Form Health Survey (SF-36). Pearson or Spearman coefficients were used to assess the relationship of paravertebral muscle function with SVA, PI, and health-related quality of life.

**Results:**

Maximal voluntary exercise (MVE) in the high-PI group was significantly lower than the MVE of both the normal- and low-PI groups (*p* < 0.05). There was no significant difference in MVE between the normal- and low-PI groups (*p* > 0.05). There was no significant difference in endurance time, SVA, ODI, RMQ, and SF-36 among the three groups. Paravertebral muscle MVE was negatively correlated with PI, SVA, ODI, and RMQ (*r* = − 0.193, − 0.210, − 0.283, − 0.277, *p* < 0.05). Endurance time of paravertebral muscle was also negatively correlated with SVA, ODI, and RMQ (*r* =  − 0.200, − 0.420, − 0.348, *p* < 0.05) and positively correlated with SF-36 (*r* = 0.245, *p* < 0.05). In addition, paravertebral muscle MVE was positively correlated with the physical functioning score of the SF-36 (*r* = 0.251, *p* < 0.05), and the endurance time of paravertebral muscle was positively correlated with the physical functioning, physical role, bodily pain, and social function scores of the SF-36 (*r* = 0.342, 0.230, 0.209, 0.256, *p* < 0.05).

**Conclusions:**

High PI may serve as a risk factor for decreased paraspinal muscle strength in patients with degenerative spinal deformities. Early and targeted exercises focusing on paraspinal muscle strength and endurance could potentially be of positive significance in slowing down the progression of sagittal imbalance, alleviating functional disorders, and increasing health-related quality of life in patients with degenerative spinal deformity.

## Introduction

Patients with degenerative spinal deformity often experience low back pain, dysfunction, and other symptoms, which seriously affect their quality of life [1]. Previous studies have shown that the sagittal vertical axis (SVA) is the spinal-pelvic parameter most closely associated with health-related quality of life (HRQL). Sagittal imbalance is a common cause of low back pain and increased dysfunction in patients with degenerative spinal deformities, which in turn affects HRQL [2].

Pelvic incidence (PI) does not change after skeletal maturity [3] and is not affected by spinal deformity, posture change, or pelvic spatial orientation [4]. A key factor affecting spinal sagittal alignment and biomechanics, PI is ideal for studies related to spinal pelvic alignment [5]. Raphaël Vialle et al*.* [6] also demonstrated the need to include PI in surgical planning. Previous studies have shown that high PI is a risk factor for degenerative spine-related disease, sagittal imbalance, and postoperative proximal junctional kyphosis.

In addition to skeletal structural parameters, paraspinal muscles are important factors in spinal stability and balance [7], and their functional decline is closely related to the occurrence and progression of degenerative spinal deformity [8, 9]. Maximal voluntary exercise (MVE) and endurance time (ET) are two important indexes for assessing muscle function [10]. The external fixation test frame has been widely used in assessment of paravertebral muscle function [11–13].

Previous studies have confirmed that paravertebral muscle strength and endurance are significantly reduced in patients with degenerative spinal deformity compared with normal healthy people of the same age [14]. However, the relationship between paravertebral muscle function and PI remains unclear. Therefore, this study investigated the relationship between PI and paravertebral muscle strength and endurance, and its effect on HRQL, in people with degenerative spinal deformity.

## Materials and methods

### General data

We selected patients with degenerative spinal deformity who were openly recruited in the Outpatient Department of Southwest Hospital (Chongqing, China) from September 2018 to June 2023, and they all signed informed consent. This study was approved by the Ethics Committee of the Southwest Hospital of Army Medical University (Approval No.: KY2020235).

We included patients 45 years or older of either gender who were diagnosed with degenerative spinal deformity (SVA ≥ 5 cm and/or Cobb > 10). The patients were divided into three groups according to PI: high (PI > 60°), normal (PI 50°–60°) and low (PI < 50°) [15].

Excluded were patients with (1) a history of other spinal dysfunction such as congenital spinal disease or spinal tuberculosis; (2) a history of spinal surgery in the past two years; (3) severe osteoporosis, lower limb joint disease, or other serious systemic diseases; (4) clear symptoms of low back pain that had an impact on daily life.

### MVE and ET tests

**Figure1:**
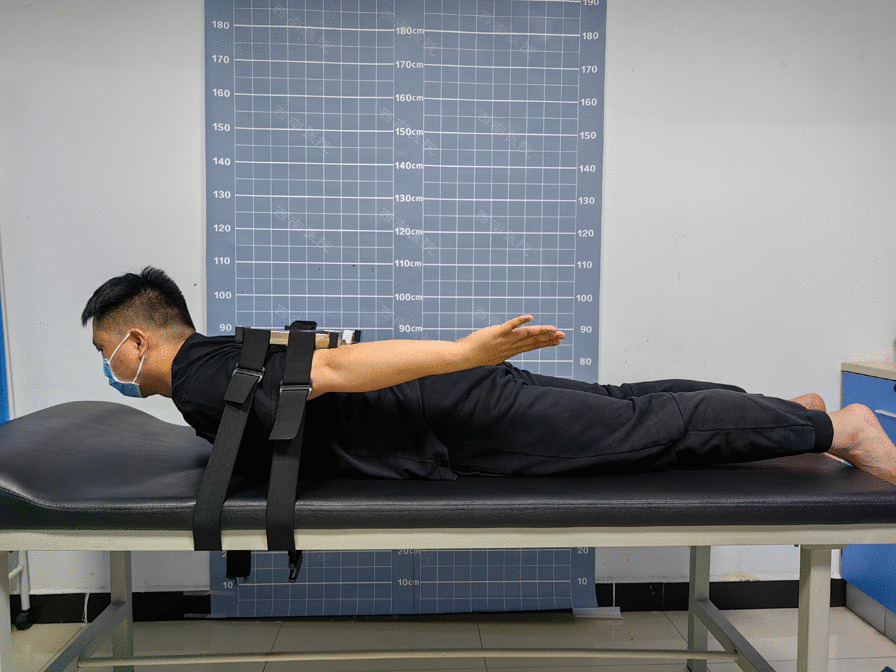
MVE test of paraspinal muscles in the prone position. Illustration: The subject extends their back and lifts their upper arm with maximum force

For the paravertebral muscle MVE test, we installed the muscle strength tester (MicroFET3) on the test support, instructed the subject to take a prone position, fixed the test support on the subject with the assistance of the staff, and then instructed the subject to extend their back and lift their upper arm with maximum force [14]. We measured three times for each subject and then took the maximum value of the three measurements as the test result (Fig. 1).

**Fig. 2:**
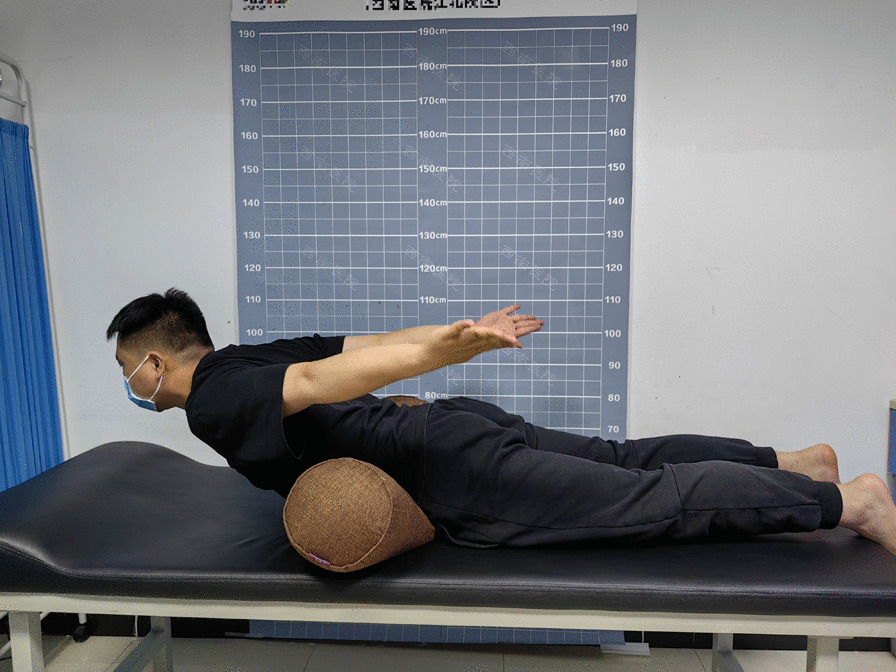
ET test of paraspinal muscles in the prone position. Illustration: The subject is placed in a prone position with a round cushion under the lower abdomen, and the duration of maintenance is recorded

For the paravertebral muscle ET test, we placed each subject prone with a round cushion under the lower abdomen and then instructed the subject to extend their back upward and raise their upper arms to lift the sternum off the test table surface [16]. We recorded how long subjects were able to maintain that position (Fig. 2).

### SVA and HRQL

All subjects underwent full-length anterolateral spine radiographs, and two spinal surgeons with more than 5 years of experience measured SVA and calculated scale scores. Subjects completed the Oswestry Disability Index (ODI), Roland–Morris questionnaire (RMQ) [17], and 36-Item Short Form Health Survey (SF-36) [18] under the guidance of the researchers.

### Statistical analysis

Statistical analysis was performed using SPSS25.0 Chinese version. Measurement data were expressed as (χ ± s). One-way analysis of variance or Kruskal–Wallis H test was used for comparison between groups. The Chi-squared test was used for constituent ratios between groups. The Pearson or Spearman correlation coefficient was used to assess the relationship between paravertebral muscle function and HRQL. For all data, *p* < 0.05 was considered statistically significant.

## Results

### General conditions

A total of 112 patients with degenerative spinal deformities were enrolled in this study, including 37 patients (5 males and 32 females) in the high-PI group, 31 (8 males and 23 females) in the normal group, and 44 (10 males and 34 females) in the low group. There were no significant differences in age, height, weight, BMI, and sex distribution among the groups (*p* > 0.05) (Table 1).

**Table 1:** Comparative results of demographics in three groups of subjects

Parameters	All	High PI	Normal PI	Low PI	P value
N	112	37	31	44	N/A
Gender (male/female)	22/90	5/32	8/23	10/34	0.427
Age (year)	64.6 ± 8.6	65.7 ± 8.4	64.6 ± 8.1	63.7 ± 9.1	0.778
Height (cm)	152.9 ± 7.5	151.4 ± 5.9	153.6 ± 8.0	153.6 ± 8.2	0.386
Weight (kg)	58.3 ± 8.7	56.2 ± 7.9	58.4 ± 8.3	60.0 ± 9.4	2.038
BMI (kg/m^2^)	24.9 ± 3.2	24.5 ± 3.0	24.6 ± 3.1	25.4 ± 3.3	0.457

*BMI* body mass index, *PI* pelvic incidence

### Comparison of paraspinal muscle function, SVA, and HRQL scores

MVE of the paravertebral muscle in the high-PI group was significantly lower than in both the normal and low groups (*p* < 0.05). There was no significant difference in MVE between normal- and low-PI groups (*p* > 0.05). ET, SVA, ODI, RMQ, and SF-36 were not significantly different among different groups (*p* > 0.05) (Table 2).

**Table 2:** Comparison results of paravertebral muscle function, SVA, and HRQL scores of three groups of subjects

Parameters	All	High PI	Normal PI	Low PI	*P* value
MVE (N)	89.5 ± 47.7	69.6 ± 36.1	97.6 ± 38.7	100.6 ± 56.8	0.009
ET (s)	34.4 ± 36.3	28.5 ± 28.8	43.9 ± 44.8	32.6 ± 34.9	0.230
SVA (cm)	4.7 ± 4.1	5.6 ± 5.0	4.0 ± 3.6	4.5 ± 3.4	0.204
ODI	44.5 ± 19.4	48.9 ± 19.5	40.1 ± 19.2	43.9 ± 19.0	0.172
RMQ	12.8 ± 6.0	13.6 ± 6.2	12.0 ± 5.5	12.8 ± 6.3	0.439
SF-36	407.1 ± 143.3	398.0 ± 144.4	437.9 ± 147.5	392.9 ± 139.4	0.425

*MVE* maximal voluntary exertion, *ET* endurance time, *SVA* sagittal–vertical axis, *PI* pelvic incidence, *ODI* Oswestry Disability Index, *RMQ* Roland–Morris questionnaire, *SF-36* 36-Item Short Form Health Survey

### Relationship of paravertebral muscle strength and endurance with SVA, PI, and HRQL

Paravertebral muscle MVE was negatively correlated with PI, SVA, ODI, and RMQ (*r* =  − 0.193, − 0.210, − 0.283, − 0.277, *p* < 0.05) and had no significant correlation with SF-36 (*r* = 0.175, *p* > 0.05). ET was negatively correlated with SVA, ODI, and RMQ (*r* =  − 0.200, − 0.420, − 0.348, *p* < 0.05), positively correlated with SF-36 (*r* = 0.245, *p* < 0.05), and had no significant correlation with PI (r =  − 0.027, *p* > 0.05) (Table 3). In addition, paravertebral muscle MVE was found to be positively correlated with the physical functioning score of the SF-36 (*r* = 0.251, *p* < 0.05), and the endurance time of paravertebral muscle exhibited positive correlations with the physical functioning, physical role, bodily pain, and social function scores of the SF-36 (*r* = 0.342, 0.230, 0.209, 0.256, *p* < 0.05) (Table 4).

**Table 3:** Relationship of paraspinal muscle function, PI and HRQL in patients with degenerative spinal deformity

Parameters	MVE		ET	
	*r*	*p*	*r*	*p*
PI	− 0.193	0.041	− 0.027	0.780
SVA	− 0.210	0.026	− 0.200	0.035
ODI	− 0.283	0.002	− 0.420	0.000
RMQ	− 0.277	0.003	− 0.348	0.000
SF-36	0.175	0.064	0.245	0.009

*MVE* maximal voluntary exertion, *ET* endurance time, *SVA* sagittal–vertical axis, *PI* pelvic incidence, *ODI* Oswestry Disability Index, *RMQ* Roland–Morris questionnaire, *SF-36* 36-Item Short Form Health Survey

**Table 4:** Correlation between paraspinal muscle function and sub items of SF-36 in patients with degenerative spinal deformity

Parameters	PF	RP	BP	GH	VT	SF	RE	MH	HT
MVE									
* r*	0.251	0.171	0.179	0.030	0.059	0.158	0.085	0.054	− 0.038
* p*	0.008	0.071	0.059	0.753	0.537	0.096	0.371	0.575	0.689
ET									
*r*	0.342	0.230	0.209	0.106	0.064	0.256	0.021	0.127	0.098
*p*	0.000	0.015	0.027	0.268	0.504	0.006	0.822	0.183	0.305

*MVE* maximal voluntary exertion, *ET* endurance time, *PF* physical functioning, *RP* role physical, *BP* bodily pain, *GH* general health, *VT* validity, *SF* social function, *RE* role emotional, *MH* mental health, *HT* health transition

## Discussion

Our study found that paravertebral muscle strength decreased significantly in patients with degenerative spinal deformities and high PI, as compared with the normal- and low-PI groups. There was no significant difference in paravertebral muscle strength between the normal- and low-PI groups. Meanwhile, we found a negative correlation between paraspinal muscle strength and PI. The reason for degeneration of muscle strength in the high-PI group may be related to steeper lumbar back inclination [5]. Disk degeneration can lead to segmental spinal instability with age, and high PI may accelerate this process, resulting in increased local disk pressure and accelerated spinal imbalance [19]. With increased spinal imbalance, more energy is required to balance the body, and the load on paravertebral muscles increases, which will eventually manifest as muscle strength attenuation.

Our results further confirmed the close relationship between the sagittal parameters represented by PI and the occurrence and development of spinal deformity [20]. No significant differences were observed in endurance among the three groups. This is probably because the participants were all over 45 years old and required less endurance to maintain the specific postures they used in daily life, resulting in similar endurance levels among all three groups.

Previous studies revealed that people with high PI are more prone to spondylolisthesis, degenerative spinal deformity, and other spinal diseases and are also at risk of sagittal imbalance [21, 22]. The results of this study showed that although there was no significant difference in SVA among the three groups, the mean SVA of the high-PI group was 5.6 cm, which reached the level of sagittal imbalance. This suggests that people with high PI are more likely to have sagittal imbalance, which is consistent with previous research results. High PI may be a result of sagittal imbalance, because sagittal imbalance of the spine increases shear forces at the sacroiliac joint plane to some extent, resulting in sacroiliac joint torsion [23].

There were no significant differences in dysfunction (as reflected in the ODI and RMQ scores) or HRQL scores among the three groups, possibly because although paravertebral muscle strength had decreased significantly, the compensatory mechanism of the spine itself could still play a crucial role [24]. Even with a slight imbalance state in the high-PI group, the attenuation of muscle strength did not reach the lowest limit that would support body weight, and thus, the impact of the deformity on the daily life of patients was not fully demonstrated.

This study focused on the potential impact of paravertebral muscle strength and endurance on SVA and HRQL. The results showed that paravertebral muscle strength and endurance were negatively correlated with sagittal imbalance, suggesting that poor muscle strength and endurance result in weaker maintenance of spinal balance. In the case of deformities, sagittal imbalance may occur when paraspinal muscle function declines to the point where it is insufficient to support the weight of the upper body, based on changes in skeletal structural parameters. Maximilian Muellner [25] et al*.* also demonstrated that increased fat infiltration in the back muscles caused a decrease in muscle strength and was one of the factors contributing to poor sagittal alignment of the spine, which is largely consistent with our results.

In addition, this study also showed that paravertebral muscle strength and endurance are closely related to lower disability scores, disability indices, and HRQL scores. Nikolaos Paramanidis [26] et al*.* found that enhancing muscle endurance training can ameliorate pain symptoms and dysfunction. Tim Schönau [27] et al*.* also showed that muscle endurance has a positive effect on enhancing lung function and improving quality of life. Takuya Miura [28] et al*.* showed a strong correlation between decreased trunk extensor strength and increased disability, which also supports our findings. Our results showed that the better the extensor function of trunk, the lower the possibility of dysfunction and pain, which had a positive impact on overall quality of life.

Based on the correlation analysis between paraspinal muscle function and the sub-items of the SF-36 scale, our findings suggest that paraspinal muscle endurance is indicative of a patient's physical functioning, physical role, bodily pain, and social function, while paraspinal muscle strength shows a positive correlation only with the dimension of physiological function. Therefore, paravertebral muscle endurance is more strongly correlated with health-related quality of life than muscle strength, and it also reflects a wider range of latitudes. Roshanravan [29] et al*.* argued that muscle endurance is a more reliable predictor than muscle strength for assessing muscle health, activity limitation, and mortality in older individuals. This is consistent with the findings of this study. Therefore, in the clinical diagnosis and treatment of patients with degenerative spinal deformity, improving the function of paravertebral muscles, in addition to correcting lumbar lordosis and sagittal imbalance, should be emphasized [30]. Early and regular exercise of lumbar back muscles, especially endurance exercise, is of great significance for preventing imbalance, improving dysfunction, and raising HRQL.

This study had several limitations. First, the effect of degree and type of deformity on HRQL was not considered. Second, this study was a single-center study, and so the results might have selection bias. Third, there was a lack of follow-up on paravertebral muscle function and HRQL, and so the dynamic changes and relationships between them need to be further clarified.

## Conclusion

High PI may serve as a risk factor for decreased paraspinal muscle strength in patients with degenerative spinal deformities. Early and targeted exercises focusing on paraspinal muscle strength and endurance could potentially have a positive impact on slowing down the progression of sagittal imbalance, alleviating functional disorders, and increasing the health-related quality of life in patients with degenerative spinal deformity.

## Data Availability

All relevant data are available from the corresponding author upon reasonable request.

## Abbreviations

BMI: Body mass index
MVE: Maximal voluntary exertion
ET: Endurance time
SVA: Sagittal vertical axis
PI: Pelvic incidence
ODI: Oswestry Disability Index
RMQ: Roland–Morris questionnaire
SF-36: 36-Item Short Form Health Survey
